# *Ab initio* molecular dynamics simulation of the effects of stacking faults on the radiation response of 3C-SiC

**DOI:** 10.1038/srep20669

**Published:** 2016-02-16

**Authors:** M. Jiang, S. M. Peng, H. B. Zhang, C. H. Xu, H. Y. Xiao, F. A. Zhao, Z. J. Liu, X. T. Zu

**Affiliations:** 1 School of Physical Electronics, University of Electronic Science and Technology of China, Chengdu 610054, China; 2 Institute of Nuclear Physics and Chemistry, Chinese Academy of Engineering Physics, Mianyang 621900, China; 3 Department of Physics, Lanzhou City University, Lanzhou 730070, China; 4 Institute of Fundamental and Frontier Sciences, University of Electronic Science and Technology of China, Chengdu 610054, China

## Abstract

In this study, an *ab initio* molecular dynamics method is employed to investigate how the existence of stacking faults (SFs) influences the response of SiC to low energy irradiation. It reveals that the C and Si atoms around the SFs are generally more difficult to be displaced than those in unfaulted SiC, and the corresponding threshold displacement energies for them are generally larger, indicative of enhanced radiation tolerance caused by the introduction of SFs, which agrees well with the recent experiment. As compared with the unfaulted state, more localized point defects are generated in faulted SiC. Also, the efficiency of damage production for Si recoils is generally higher than that of C recoils. The calculated potential energy increases for defect generation in SiC with intrinsic and extrinsic SFs are found to be higher than those in unfaulted SiC, due to the stronger screen-Coulomb interaction between the PKA and its neighbors. The presented results provide a fundamental insight into the underlying mechanism of displacement events in faulted SiC and will help to advance the understanding of the radiation response of SiC with and without SFs.

Cubic silicon carbide (3C-SiC) exhibits lots of superior electronic and physical properties such as high electron mobility, saturated electron drift velocity, high corrosion resistance, favorable chemical inertness and small neutron absorption cross-section^1^,^2^,^3^,^4^,^5^. It is thus desirable for power switching device applications and is often utilized under harsh environment such as high temperature and high pressure. Due to the superior physical properties of 3C-SiC, it has been considered as a vital component in nuclear applications. For example, 3C-SiC has been considered as an inert-matrix material for water-cooled reactors to burn minor actinides or Pu^4^, a structural material for fusion power reactors^6^, a cladding material for nuclear fuel, and a structural material for the reactor core in high temperature gas-cooled reactors^7^. It is therefore of critical importance to understand the phase stability of 3C-SiC under irradiation and explore the way to enhance its radiation tolerance.

In the past decades, a great number of experimental and theoretical studies have been carried out to investigate the radiation damage effects of SiC^8^,^9^,^10^,^11^,^12^. Inui *et al.* have found that crystalline-to-amorphous transition in single crystalline silicon carbide (sc-SiC) can be induced by electron irradiation at temperatures around 300 K^13^,^14^. Several ion irradiation studies on nano-crystalline silicon carbide (nc-SiC) have also been reported and it has been suggested that reducing the grain size may improve the mechanical properties of sc-SiC^15^,^16^. Recently, Zhang *et al.* compared the radiation tolerance of sc-SiC and nc-SiC by employing 550 keV Si^+^ ion irradiation, who found that the sc-SiC readily undergoes irradiation-induced structural amorphization, whereas the nc-SiC with a high-density of stacking faults (SFs) exhibits more than an order of magnitude increase in radiation resistance^17^. Jamison *et al.* studied the crystalline-to-amorphous transition in nc-SiC using 1.25 MeV electron irradiation, and found that the nc-SiC has an increased dose to amorphization, as compared with the sc-SiC. They proposed that the addition of a high density of grain boundaries, grain texture, and the presence of SFs may all contribute to the enhanced radiation tolerance^18^. Theoretically, the density functional theory (DFT) method has been employed to study the fundamental properties of SiC containing intrinsic stacking faults (ISFs) and (or) extrinsic stacking faults (ESFs). Umeno *et al.* have investigated the SF formation energy and the stress-strain relationship induced by the SF formation^19^. Oda *et al.* have studied the formation energy and electronic structure of SiC with ISFs^20^. Jamison *et al.* have investigated how the SFs influence the dose to amorphization in SiC and found that the energy barriers for Si interstitial migration and the rate-limiting defect recovery reaction are reduced by the existence of SFs^18^. In spite of these extensive studies, the dynamic processes for defect generation in SF-contained SiC at an atomic level have not been revealed yet. Besides, the origin of the enhanced radiation tolerance caused by the SF formation needs to be further explored.

In recent years, the *ab initio* molecular dynamics (AIMD) method, in which the interatomic potential is obtained by electronic structure calculations rather than empirical fitting, has been demonstrated to be a powerful tool in simulating the displacement events in ceramic materials^21^,^22^,^23^,^24^,^25^. It has been revealed that physical parameters like threshold displacement energy can be determined with *ab initio* accuracy, and new mechanism for defect generation and new defective states that are different from classical molecular dynamics (MD) can be predicted. In particular, the role of charge transfer during the dynamic process of recoil events can be elucidated. In this study, the AIMD method is employed to study the low-energy recoil events of 3 C-SiC with SFs. Our main aims are (1) to investigate the defect generation mechanism and defect distribution in SiC with SFs; (2) to compare the response of unfaulted and faulted SiC to low energy radiation; and (3) to explore the origin of the difference in the radiation susceptibility between SF-contained SiC and the unfaulted state.

## Results and Discussion

### Ground-state properties for bulk SiC and stacking fault formation energy

To test the pseudopotentials of Si and C, the lattice constant and cohesive energy for bulk SiC are first calculated and compared with experimental and other theoretical values in Table 1. It is shown that our results are in excellent agreement with experiments and are comparable with other theoretical values^26^,^27^. The defect formation energy, which is defined by *E_f_* = *E_def_* - *E_undef_* + 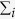
∆*n_i_µ_i_*^18^, is calculated for ISFs and ESFs. Here, 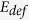 is the energy of the faulted supercell, 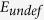 is the energy of the unfaulted supercell, 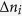 is the change in the number of species 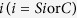 and 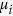 is the chemical potential of species *i*. The chemical potentials of silicon (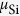) and carbon (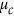) obey the following criteria: 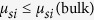, 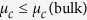 and 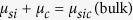, where 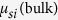 and 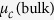 are the chemical potentials of bulk Si and diamond, respectively, and 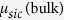 is the total energy of bulk SiC^28^. The SF formation energies are calculated under both carbon-rich (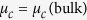 and 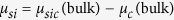) condition and silicon-rich (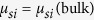 and 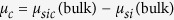) condition. For the three types of ISFs, the calculated formation energies under both conditions are found to be nearly identical to each other, i.e., ~−7.8 mJ/m^2^, which agrees well with the value of −3.4 mJ/m^2^ reported by Käckell *et al.*^29^. Umeno *et al.* have determined the formation energy to be 9.82 mJ/m^2^ for a single-layer ISF by DFT method^19^, which differs greatly from our calculations. Such discrepancy mainly results from the differences in the size of the supercell. In our calculations the supercell for SiC with ISFs consists of 256 atoms, while the supercell employed by Umeno *et al.* consists of only 10 atoms^19^. Similarly, the three types of ESFs exhibit very similar stability. The SF formation energy is calculated to be ~1.7 mJ/m^2^, which differs a lot from the value of −28 mJ/m^2^ reported by Käckell *et al.*^29^ This may be due to the differences in the employed exchange-correlation potentials. Käckell *et al.* carried out the calculations within the framework of local-density approximation, while our calculations are performed by the generalized gradient approximation. Comparing with the experimental value of 2.5 mJ/m^2^ 30, we find that our calculated value of ~1.7 mJ/m^2^ is in good agreement with experiments.

### Threshold displacement energies for C and Si recoils in unfaulted and faulted SiC

The critical physical parameter for estimating damage production rates under electron, neutron, and ion irradiation and predicting the defect profile is the threshold displacement energy (E_d_), which can be defined as the minimum transferred kinetic energy for the primary knock-on atom (PKA) to be permanently displaced from its lattice site and form stable defects^12^. In the past several years, the E_d_ values in a number of semiconductors and ceramic materials have been investigated employing the AIMD method^23^,^25^. In order to explore how the existence of SFs affects the radiation response of SiC, we first calculate the E_d_s for C and Si recoils in unfaulted SiC along the 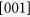 and 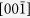 directions, which are perpendicular to the SiC(111) plane and correspond to the 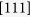 and 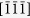 directions in bulk SiC, respectively. A comparison of our results with other theoretical values is provided in Table 2. The E_d_s for C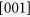 and C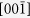 are determined to be 19 and 47.5 eV, respectively. For Si recoils, the E_d_ values are calculated to be 95 eV for the 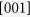 direction and 63 eV for the 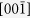 direction. It is shown that our results are in good agreement with the results reported by Gao *et al.*^12^. Comparing our results with the classical MD simulation carried out by Devanathan and Weber^31^, we find that the E_d_s obtained by the AIMD method are generally much smaller, except for the case of Si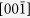. This may be due to the fact that charge transfer that occurs during the recoil events is taken into account by the AIMD method while not considered in the classical MD simulations^32^.

**Figure 1:**
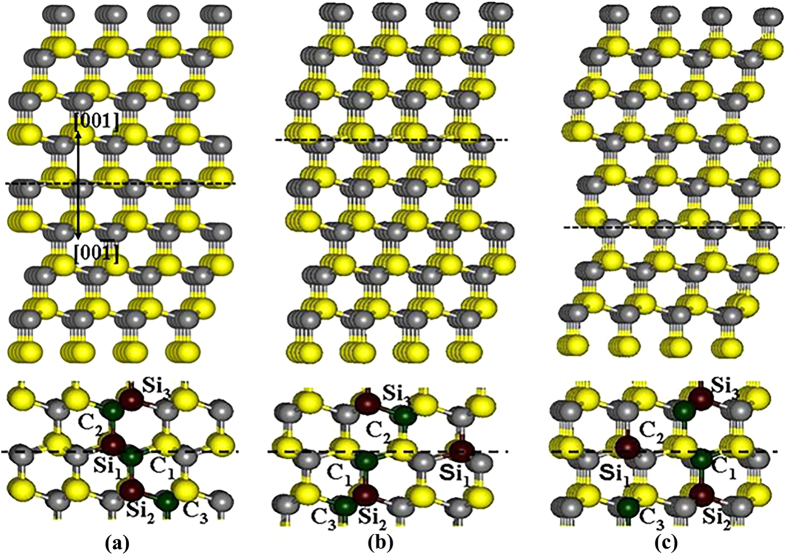
Illustration of schematic view of SiC containing intrinsic SFs with (**a**) (ABC)(AC)(ABC); (**b**) (ABC)(AB)(ABC); (**c**) (ABC)(BC)(ABC) stacking sequences.

**Figure 2:**
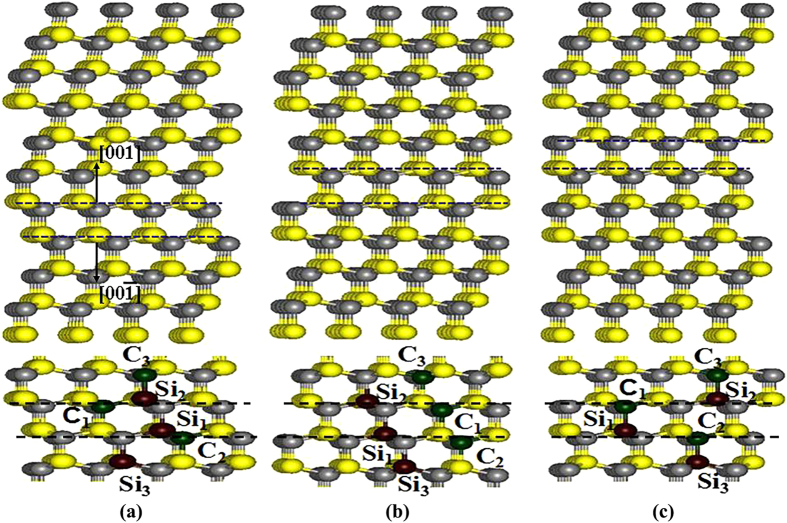
Illustration of schematic view of SiC containing extrinsic SFs with (**a**) (ABC)(BABC)(ABC); (**b**) (ABC)(ACBC)(ABC); (**c**) (ABC)(ABAC)(ABC) stacking sequences.

The calculated E_d_s for C and Si PKAs in SiC with ISFs and ESFs (see Figs 1 and 2) are summarized in Table 3. As for C recoils around the ISFs, it is found that along both the [001] and 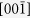 directions the E_d_ values for C_3_ PKAs are generally larger than those for C_1_ and C_2_ PKAs. In the case of C recoils around the ESFs, the E_d_ values for C_1_ PKAs are the highest for both [001] and 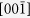 directions. Obviously, the three types of C PKAs that have different interlayer spacing from the SFs exhibit different tolerance to irradiation. Similar phenomenon is also observed for Si recoils, for which the E_d_s in several cases are larger than 150 eV, i.e., the PKA is not permanently displaced at energy up to 150 eV. It is found that the three types of Si recoils around the SFs are affected remarkably and exhibit different E_d_ values. Comparing the E_d_s for C and Si PKAs, we find that generally considerably higher energies are needed for displacing the Si PKAs than those for displacing the C PKAs, similar to the cases in bulk SiC^12^. These results show that the radiation susceptibility of the C and Si atoms around the SFs is affected significantly by the existence of SFs.

In this study, the weighted average E_d_ values are calculated to be 52.1 eV for C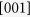, 59.5 eV for C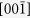, >99.6 eV for Si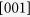 and >122.1 eV for Si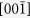 in SiC containing ISFs. As for the PKAs in SiC with ESFs, the average E_d_ values are calculated to be 37.8, 71.6, >128.4 and >88.1 eV for C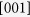, C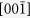, Si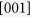 and Si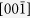, respectively. Comparing these results with the values of 19, 47.5, 95 and 63 eV for C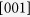, C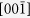, Si
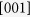
and Si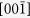 in unfaulted SiC, respectively, we find that the E_d_ values in faulted SiC are generally larger. The maximum energy transferred to an atom can be expressed as 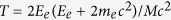 under electron irradiation, where *E*_*e*_ is the incident energy, *m*_*e*_ is the electronic mass, *M* is the atomic mass and *c* is the velocity of light^33^. Assuming 300 keV electrons incident on SiC, the maximum energy transferred to Si and C atom are 59.5 and 71 eV, respectively. Our finding that the E_d_ values of C and Si recoils are increased by the existence of SFs, therefore, suggests that SiC with SFs is less susceptible to low energy irradiation. This is consistent with the experiments carried out by Zhang *et al.*^17^ and Jamison *et al.*^18^. Employing 550 keV Si^+^ ion irradiation, Zhang *et al.* investigated the radiation tolerance of SiC with and without SFs, and found that the SiC with a high-density of SFs exhibits more than an order of magnitude increase in radiation resistance^17^. Jamison *et al.* studied the crystalline-to-amorphous transition in SiC using 1.25 MeV electron irradiation, and also found that SiC with ISFs or ESFs behaves more robustly under irradiation environment^18^.

### Defect distribution in unfaulted and faulted SiC

The defects created by C and Si PKAs in recoil events are summarized in Tables 4 and 5, respectively. In the case of C PKAs, the defects created in unfaulted SiC mainly consist of the carbon vacancy (C_vac_) and carbon interstitial (C_int_), as shown in Table 2, which agrees well with the results reported by Gao *et al.*^12^. Comparing the damage end states created by different carbon recoils in SiC with ISFs, we find that C_2_ PKAs show similar defect distribution to unfaulted SiC. However, the Frenkel pair separation (d_FP_) between the carbon vacancy and carbon interstitial is somewhat different. As compared with the d_FP_ of 4.02 Å for C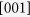 in unfaulted SiC, the separations are 3.83, 4.38, 4.42 Å for C_2_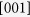 with (ABC)(AC)(ABC), (ABC)(AB)(ABC) and (ABC)(BC)(ABC) stacking sequences, respectively. For C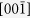 in unfaulted SiC, the Frenkel pair distance is 1.67 Å, which is much smaller than the values of 2.45, 2.51 and 2.50 Å for C_2_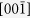 in SiC with (ABC)(AC)(ABC), (ABC)(AB)(ABC) and (ABC)(BC)(ABC) stacking sequences, respectively. These results suggest that in spite of similar damage end states, the pathway for defect generation should be different. For C_1_ and C_3_ PKAs, the defect generations are more complex. Besides the C FPs, the neighboring Si atoms of the C PKAs are involved in the recoil events, resulting in the additional formation of Si occupying the lattice C site (Si_C_), C occupying the lattice Si site (C_Si_), and (or) Si interstitials (Si_int_). As for C PKAs in SiC with ESFs, the damage end states generated by C_2_ and C_3_ PKAs are generally similar to those for C PKAs in unfaulted SiC, except for C_2_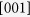 with (ABC)(ABAC)(ABC) arrangement. In this case, the neighboring Si atom is ejected along the 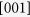 direction and then rebounds along the opposite direction to occupy the original site of C PKA. In the meantime, the vacant Si site is occupied by the C PKA, leading to the formation of Si_C_ and C_Si_ antisite defects. The mechanism for defect generation in C_1_ recoil events is different from that in C_2_ and C_3_ recoils events. The C_1_ PKA collides directly with its nearest-neighboring Si atom along the 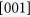 (or 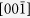) direction and occupies the Si lattice site to form C_Si_ defect. The struck Si atom receives sufficient energy and moves along the 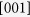 (or 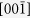) direction to replace its neighboring C atom. The replaced C atom then moves away from its lattice site to form stable carbon interstitial. As a result, the final defect structure consists of a C FP, a Si_C_ antisite defect and a C_Si_ antisite defect. Our calculations show that the total defect number generated by C PKAs in faulted SiC is generally not less than that in unfaulted SiC.

In unfaulted SiC, the defects created by Si PKAs under low energy irradiation are one C FP, two Si_C_ and two C_Si_ antisite defects for Si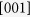, and one Si FP for Si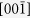. Comparing the damage end states created by different Si recoils in SiC with ISFs, we find that Si_3_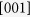 show similar defect distribution to unfaulted SiC, whereas Si_1_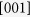, Si_2_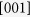 and Si_2_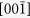 recoil events exhibit different character. For Si_1_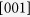, the damage end states consist of three Si_C_, two C_Si_, one Si vacancy and one C interstitial. In the case of Si_2_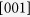, only two antisite defects (and one C FP for SiC with (ABC)(AB)(ABC) stacking sequence) are generated. Regarding Si_2_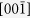, the created defects are one Si_C_, one Si_vac_ and one C_int_ for SiC with (ABC)(AC)(ABC) and (ABC)(AB)(ABC) stacking sequences, and one Si FP for SiC with (ABC)(BC)(ABC) stacking sequence.

As for Si PKAs in SiC with ESFs, only one Si FP is generated by Si_2_ along the 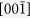 direction and two antisite defects are generated by Si_2_ along the 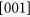 direction in SiC with (ABC)(ACBC)(ABC) stacking sequence. For the Si_3_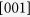, the damage end states in SiC with (ABC)(BABC)(ABC) and (ABC)(ACBC)(ABC) arrangements are similar to those in unfaulted SiC, whereas the recoil events in SiC with (ABC)(ABAC)(ABC) stacking sequence show different end states, i.e., two Si_C_, one C_Si_, one Si vacancy and one C interstitial. In the case of Si_3_ PKAs along the 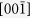 direction, the defect generation are relatively simple, as indicated by C_Si_ + Si_C_ for (ABC)(BABC)(ABC), Si FP for (ABC)(ABAC)(ABC), and Si_C_ + C_int_ + Si_vac_ for (ABC)(ACBC)(ABC) stacking sequences.

**Figure 3:**
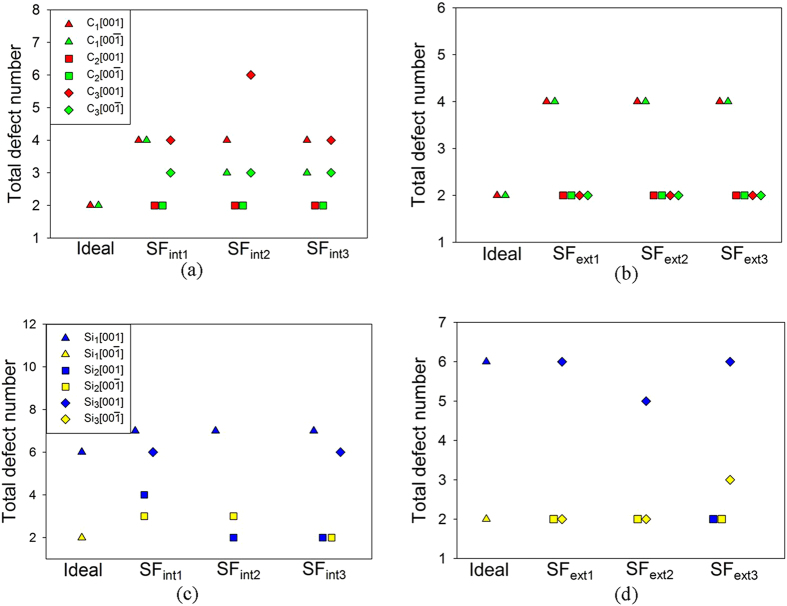
Total defect number created by (**a**) C PKAs in SiC with intrinsic SFs; (**b**) C PKAs in SiC with extrinsic SFs; (**c**) Si PKAs in SiC with intrinsic SFs; and (**d**) Si PKAs in SiC with extrinsic SFs. Here, the SF_int1_, SF_int2_ and SF_int3_ represent intrinsic SFs with (ABC)(AC)(ABC), (ABC)(AB)(ABC) and (ABC)(BC)(ABC) atomic arrangements, respectively, and the SF_ext1_, SF_ext2_ and SF_ext3_ represent extrinsic SFs with (ABC)(BABC)(ABC), (ABC)(ABAC)(ABC) and (ABC)(ACBC)(ABC) atomic arrangements, respectively.

The total defect number created by C and Si PKAs in faulted SiC is illustrated in Fig. 3. It is found that the Si PKAs are generally more efficient in damage production than C PKAs^34^. Weber *et al.* have calculated the efficiency of damage production for C, Si and Au PKAs over the energy range from 0.1 to 400 keV using a modified version of the stopping and range of ions in matter (SRIM) code^34^. They suggested that the total damage efficiency for C PKAs is much lower than that for Si PKAs at low damage energies^34^, which is consistent with our results. Comparing the defects generated by Si PKAs in faulted SiC, we find that the defect configurations are similar, i.e., antisite defect, Si FP and C FP. Besides, the defect number for different Si PKA along a certain incident direction is nearly identical to each other. Zhang *et al.* applied the MD method to study the defect production in sc-SiC, SiC with a high density of ESFs and SiC with a high density of ISFs, and found that there are no great difference among the three simulation cells in defect number and configurations^17^. In the meantime, the distribution of created defects in faulted SiC is shown to be very localized. These results agree well with the study of radiation tolerance of sc-SiC and SiC with SFs performed by Zhang *et al.*, in which it was found that the existence of SFs leads to more localized point defect production^17^. Comparing the different defect configurations for unfaulted and faulted SiC, we find that antisite defects are the most common defects in faulted SiC, whereas in unfaulted SiC the FPs are dominant. The defect generation in sc-SiC and nc-SiC with a grain size smaller than 12 nm have been investigated by Gao *et al.* using the MD method, in which the kinetic energies of 10 keV for PKA were simulated. They also found that in nc-SiC the antisite defects are more than other defects, in contrast to those produced in sc-SiC, where the dominant defects are FPs^35^.

### Origin of the difference in the radiation response between unfaulted and faulted SiC

**Figure 4:**
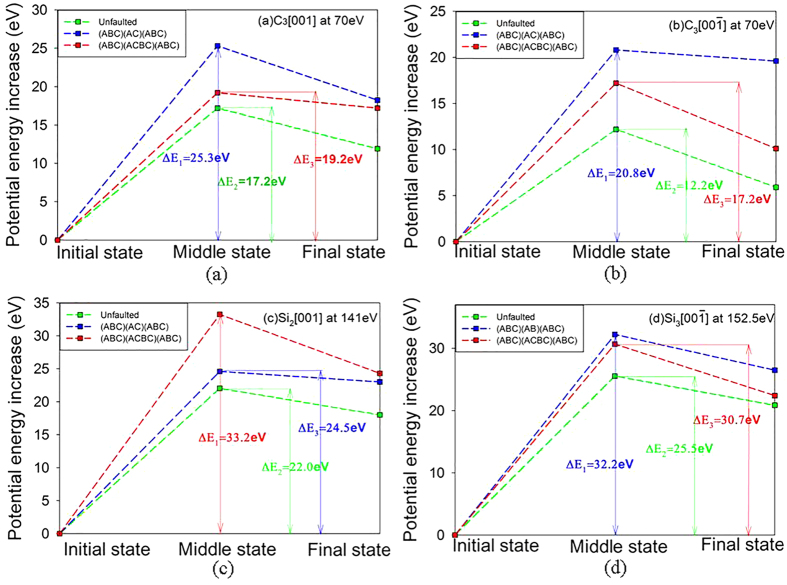
The calculated potential energy increase for (**a**) C_3_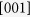 at 70 eV; (**b**) C_3_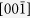 at 70 eV; (**c**) Si_2_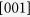 at 141 eV; and (**d**) Si_3_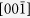 at 152.5 eV.

Jamison *et al.* studied the energetics of point defects near the SFs and found that the critical migration and reaction energies are reduced significantly enough to enhance the amorphization resistance by increasing the probability of point defect recombination and annihilation^18^. To explore the origin of the difference in the radiation response behavior of unfaulted and faulted SiC, we further analyze the potential energy increase for stable defect formation in the recoil events of C_3_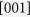 at 70 eV, C_3_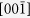 at 70 eV, Si_2_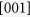 at 141 eV and Si_3_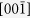 at 152.5 eV. As illustrated in Fig. 4(a), the maximum potential energy increases for C_3_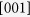 are 25.3, 19.2 and 17.2 eV for SiC with (ABC)(AC)(ABC) arrangement, SiC with (ABC)(ACBC)(ABC) arrangement and unfaulted SiC, respectively. In the case of C_3_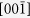, the maximum potential energy increases are 20.8, 17.2 and 12.2 eV, corresponding to SiC with (ABC)(AC)(ABC), (ABC)(ACBC)(ABC) arrangements and unfaulted SiC, respectively. The situation in Si_2_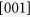 at 141 eV and Si_3_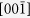 at 152.5 eV are very similar to those in C recoil events, i.e., the maximum potential energy increases for SiC with SFs are always larger than those for unfaulted SiC. The maximum potential energy increases represent the maximum in screen-Coulomb interactions between PKAs and one or more atomic nuclei on lattice or defect sites, similar to classical two-body interaction^33^. Our results show that the introduction of SFs leads to greater maximum potential energy increase than unfaulted state, i.e., stronger interaction due to more effective screening of Coulomb force between PKA and its neighbors exist in faulted SiC, which may increase the energy barrier for defect generation. Consequently, a greater kinetic energy is necessary to overcome the larger energy barrier for defect generation, corresponding to the larger threshold displacement energies for C and Si PKAs in faulted SiC than those in unfaulted SiC. Another finding is that the maximum potential energy increases for Si PKAs are generally larger than those for C PKAs, which is consistent with our results that generally considerably higher energies are needed for displacing the Si PKAs than those for displacing the C PKAs.

## Conclusions

In summary, low-energy recoil events in unfaulted and faulted SiC have been investigated by *ab initio* molecular dynamics method based on density functional theory. The threshold displacement energies are shown to be dependent on the interlayer spacing between the PKA and the SFs. The weighted average E_d_ values are calculated to be 52.1 eV for C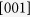, 59.5 eV for C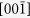, >99.6 eV for Si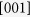 and >122.1 eV for Si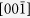 in SiC with ISFs. As for SiC containing ESFs, the average E_d_ values are 37.8, 71.6, >128.4 and >88.1 eV for C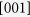, C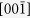, Si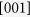 and Si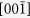, respectively. As compared with the E_d_ values in unfaulted SiC, the E_d_ values in faulted SiC are generally larger, i.e., the PKAs in faulted SiC are more difficult to be displaced, which may enhance the radiation tolerance of SiC, agreeing well with the recent experiments. In the meantime, the defect generation mechanism for C and Si PKAs in faulted SiC is generally more complex and the defect contribution is very localized. The most common defect configurations in faulted SiC are antisite defects, whereas the Frenkel pairs are dominant in unfaulted SiC. Potential energy increase analysis shows that the existence of SFs increases the energy barrier for defect generation, i.e., the C and Si primary knock-on atoms in faulted SiC need to overcome higher energy barrier than those in unfaulted SiC to generate defects.

## Methods

All the calculations are carried out using the Spanish Initiative for Electronic Simulations with Thousands of Atoms (SIESTA) code. The norm-conserving Troullier-Martins pseudopotential^36^ are employed to determine the interaction between ions and electrons and the exchange-correlation potential is described by the generalized gradient approximation parameterized by Perdew, Burke and Ernzerhof^37^. The valence wave functions are expanded by a basis set of localized atomic orbitals and single-ζ basis sets are employed, with a K-point sampling of 1 × 1 × 1 in the Brillouin zone and a cutoff energy of 90 Ry. In the literature, Zhang *et al.*^17^ and Lin *et al.*^38^ have reported that the SFs lie in the (111) plane of SiC. Hence, both the ISFs and ESFs investigated in this study are created based on the 3C-SiC(111) plane. For SiC with ISFs and ESFs, the supercell consists of 256 and 320 atoms, respectively. Three types of ISFs, i.e., (ABC)(AC)(ABC), (ABC)(AB)(ABC) and (ABC)(BC)(ABC) and three types of ESFs, i.e., (ABC)(BABC)(ABC), (ABC)(ABAC)(ABC) and (ABC)(ACBC)(ABC), as shown in Figs 1 and 2, have been considered. To simulate the low energy recoil events, three types of Si or C on the boundary of the SFs, as denoted in Figs 1 and 2, are selected as PKA and a certain amount of kinetic energy is provided along the direction perpendicular to the SiC(111) surface, i.e., 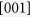 and 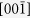. The simulations are conducted with a NVE ensemble and a variable time step scheme is employed to avoid the instability of the system.

## Additional Information

**How to cite this article**: Jiang, M. *et al.* *Ab initio* molecular dynamics simulation of the effects of stacking faults on the radiation response of 3C-SiC. *Sci. Rep.* **6**, 20669; doi: 10.1038/srep20669 (2016).

## Figures and Tables

**Table 1:** Calculated lattice constant and cohesive energy for bulk SiC.

	Lattice constant (Å)	Cohesive energy (eV/atom)
Our calc.	4.37	6.40
Other calc.	4.361^a^, 4.45^b^	6.66^a^
Exp.	4.36^a^	6.34^a^

^a^ref. 27.

^b^ref. 26.

**Table 2:** Calculated threshold displacement energies (E_d_s) and the associated defect types for C and Si recoil events along the direction normal to the SiC(111) surface.

		E_d_(eV)	Defect type
C 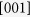	Our Calc.	19	C_vac_ + C_int_
	Other MD	20.5^a^, 30.0^b^	C_vac_ + C_int_
C 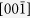	Our Calc.	47.5	C_vac_ + C_int_
	Other MD	47.5^a^, 71.0^b^	C_vac_ + C_int_
Si 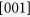	Our Calc.	95	2Si_C_ + 2 C_Si_ + C_vac_ + C_int_
	Other MD	105^a^, 108.0^b^	Si_vac_ + Si_int_
Si 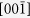	Our Calc.	63	Si_vac_ + Si_int_
	Other MD	62^a^, 38.0^b^	Si_vac_ + Si_int_

C_vac_: C vacancy; C_int_: C interstitial; Si_vac_: Si vacancy; Si_int_: Si interstitial; C_Si_: C occupying the lattice Si site; Si_C_: Si occupying the lattice C site.

^a^ref. 12.

^b^ref. 31.

**Table 3:** Calculated threshold displacement energies (E_d_s) for C and Si in SiC with intrinsic stacking faults (ISFs) and extrinsic stacking faults (ESFs).

	Stacking sequence	Direction	E_d_ (eV)					
			C_1_	C_2_	C_3_	Si_1_	Si_2_	Si_3_
ISFs	(ABC)(AC)(ABC)	[001]	64	40	**68.5**	69	**131**	80.5
		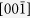	57	58	**63**	**>150**	65	**>150**
	(ABC)(AB)(ABC)	[001]	64	19.5	**67**	68.5	128	**>150**
		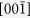	54	58	**66.5**	**>150**	66	**>150**
	(ABC)(BC)(ABC)	[001]	**64**	19.5	62	68.5	**130**	70.5
		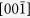	56	57.5	**65**	**>150**	68	**>150**
ESFs	(ABC)(BABC)(ABC)	[001]	**67**	19.5	19.5	**>150**	**>150**	87.5
		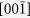	**112.5**	46.5	50.5	**>150**	49	65
	(ABC)(ABAC)(ABC)	[001]	**66.5**	19	19.5	**>150**	**>150**	88.5
		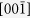	**120.5**	50	48.5	**>150**	49	64
	(ABC)(ACBC)(ABC)	[001]	**67**	19.5	42.5	**>150**	141	88.5
		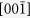	**115.5**	49	51	**>150**	49	67

The maximum E_d_ values for C and Si PKAs in each SiC with SFs are indicated in bold.

**Table 4:** Defect configurations for C recoil events in SiC with intrinsic stacking faults (ISFs) and extrinsic stacking faults (ESFs).

	Stacking sequence	Direction	Defect type		
			C_1_	C_2_	C_3_
ISFs	(ABC)(AC)(ABC)	[001]	C_Si_ + Si_C_ + C_int_ + C_vac_	C_vac_ + C_int_	C_Si_ + Si_C_ + C_int_ + C_vac_
		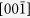	C_Si_ + Si_C_ + C_int_ + C_vac_	C_vac_ + C_int_	C_vac_ + C_Si_ + Si_int_
	(ABC)(AB)(ABC)	[001]	C_Si_ + Si_C_ + C_int_ + C_vac_	C_vac_ + C_int_	2Si_C_ + 2 C_Si_ + C_int_ + C_vac_
		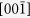	C_vac_ + C_Si_ + Si_int_	C_vac_ + C_int_	C_vac_ + C_Si_ + Si_int_
	(ABC)(BC)(ABC)	[001]	C_Si_ + Si_C_ + C_int_ + C_vac_	C_vac_ + C_int_	C_Si_ + Si_C_ + C_int_ + C_vac_
		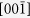	C_vac_ + C_Si_ + Si_int_	C_vac_ + C_int_	C_vac_ + C_Si_ + Si_int_
ESFs	(ABC)(BABC)(ABC)	[001]	C_Si_ + Si_C_ + C_int_ + C_vac_	C_vac_ + C_int_	C_vac_ + C_int_
		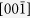	C_Si_ + Si_C_ + C_int_ + C_vac_	C_int_ + C_vac_	C_vac_ + C_int_
	(ABC)(ABAC)(ABC)	[001]	C_Si_ + Si_C_ + C_int_ + C_vac_	C_Si_ + Si_C_	C_vac_ + C_int_
		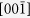	C_Si_ + Si_C_ + C_int_ + C_vac_	C_vac_ + C_int_	C_vac_ + C_int_
	(ABC)(ACBC)(ABC)	[001]	C_Si_ + Si_C_ + C_int_ + C_vac_	C_vac_ + C_int_	C_vac_ + C_int_
		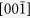	C_Si_ + Si_C_ + C_int_ + C_vac_	C_vac_ + C_int_	C_vac_ + C_int_

The denotations for the defect type are the same as those in Table 2.

**Table 5:** Defect configurations for Si PKA recoil events in SiC with intrinsic stacking faults (ISFs) and extrinsic stacking faults (ESFs).

	Stacking sequence	Direction	Defect type		
			Si_1_	Si_2_	Si_3_
ISFs	(ABC)(AC)(ABC)	[001]	3Si_c_ + 2 C_Si_ + Si_vac_ + C_int_	Si_C_ + C_Si_ + C_vac_ + C_int_	2Si_C_ + 2 C_Si_ + C_vac_ + C_int_
		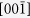	–	Si_C_ + Si_vac_ + C_int_	–
	(ABC)(AB)(ABC)	[001]	3Si_c_ + 2 C_Si_ + Si_vac_ + C_int_	Si_C_ + C_Si_	–
		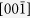	–	Si_C_ + Si_vac_ + C_int_	–
	(ABC)(BC)(ABC)	[001]	3Si_c_ + 2 C_Si_ + Si_vac_ + C_int_	C_Si_ + Si_C_	2Si_C_ + 2 C_Si_ + C_int_ + C_vac_
		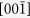	–	Si_int_ + Si_vac_	–
ESFs	(ABC)(BABC)(ABC)	[001]	–	–	2Si_C_ + 2 C_Si_ + C_int_ + C_vac_
		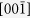	–	Si_int_ + Si_vac_	C_Si_ + Si_C_
	(ABC)(ABAC)(ABC)	[001]	–	–	2Si_C_ + C_Si_ + Si_vac_ + C_int_
		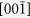	–	Si_int_ + Si_vac_	Si_int_ + Si_vac_
	(ABC)(ACBC)(ABC)	[001]	–	C_Si_ + Si_C_	2Si_C_ + 2 C_Si_ + C_int_ + C_vac_
		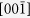	–	Si_int_ + Si_vac_	Si_C_ + C_int_ + Si_vac_

The denotations for the defect type are the same as those in Table 2.
